# Editorial: Taking a hands-on approach: current perspectives on the effect of hand position on vision

**DOI:** 10.3389/fpsyg.2015.01231

**Published:** 2015-08-18

**Authors:** Christopher C. Davoli, Philip Tseng

**Affiliations:** ^1^Department of Psychology, Central Michigan UniversityMt. Pleasant, MI, USA; ^2^Graduate Institute of Humanities in Medicine, Taipei Medical UniversityTaipei, Taiwan; ^3^Brain and Consciousness Research Center, Shuang-Ho HospitalNew Taipei City, Taiwan

**Keywords:** embodied cognition, multisensory integration, perception and action, affordance, proprioception

Over the past 10 years, perception scientists have uncovered a surprising connection between people's vision and their hands. There is now compelling evidence that how people perceive, attend to, think about, and remember visual information depends on how close they have their hands to that information. With their hands near, people perform figure-ground assignment more efficiently, parse temporally adjacent events more precisely, and hold more information in visual working memory. Near their hands, people also detect sudden visual onsets more quickly, but search through arrays of items more slowly, and take longer to switch between different ways of interpreting the same perceptual content (e.g., “seeing the forest” vs. “seeing the trees”). These are but some of the ways in which visual processing changes when people's hands are in proximity of viewed information—a host of effects that we refer to here, collectively, as hand-altered vision (HAV).

The first decade of research into HAV has generated a substantial amount of new knowledge, which we recently reviewed in contemporaneous papers (Tseng et al., 2012; Brockmole et al., 2013). We subsequently established this Research Topic as a bridge to the next era of HAV research, through which we aimed to gather perspectives from across the research literatures on human action and peripersonal space representation. All told, the work here consists of 12 articles from 34 researchers who represent 23 institutions worldwide. Thanks to the efforts of our contributors, our scientific understanding of HAV has progressed along several major channels.

## Visual attention near the hands: mechanisms, modulating factors, and new directions

The research literature on HAV began in earnest with two key findings about visual attention. First, people tend to prioritize their attention to visual signals in near-hand space over other locations (*prioritization effect*). Second, people are slower to disengage their attention from locations near their hands (*disengagement effect*). Considering the practical implications and potential applications of these effects, there has been a critical need for research into how and under what conditions hand-altered attention works. The following studies make considerable strides toward meeting that need.

We begin with a landmark study into the neurophysiological bases of near-hand effects on attention. Utilizing a combination of behavioral methods and neuroimaging (electroencephalography), Reed et al. (2013) found converging evidence for the existence of a prioritization effect and a disengagement effect during early and later stages of processing, respectively. Moreover, by capturing the neural signatures of these effects in the same perceptual episode, this work puts forth the most precise and comprehensive picture to date of hand-altered attention as it unfolds in real-time.

We also gain new insight into the factors that modulate hand-altered attention. To start, we learn that grasp posture makes a difference for the prioritization effect. As Thomas' work (2013) shows, people are more likely to prioritize locations near their hands when their hand posture affords a task-appropriate action (Thomas, 2013). We also learn that the disengagement effect may not be immune to one's recent postural history. Evidence of this comes from Schultheis and Carlson (2013). The more hand positions they tested within a single experimental session (visual search), the less likely their participants were to exhibit the typical disengagement effect. Finally, we see that different components of hand-altered attention may not necessarily be modulated by the same factors. Preliminary evidence of this stems from Vatterott and Vecera's study (2013), in which participants did not exhibit a prioritization effect but did show a disengagement effect during visual search (Vatterott and Vecera, 2013). Although the reasons for this dissociation are not yet clear, the results are consistent with the notion that prioritization of near-hand space was disrupted by certain unique features of the testing paradigm.

To conclude this section, we are pleased to present works that push the study of hand-altered attention into contexts considerably more complex than is typical in HAV research. Nearly, every study of hand-altered attention to date has involved participants seated at a computer while holding their hands at a fixed location either near to or far from the test stimuli. By contrast, many real-world tasks of visual attention involve the coordinated use of both hands in different states of activity, as when slicing a cucumber or using a smartphone. How do people prioritize their attention in scenarios like these? Thanks to Festman et al. (2013), we now have a clearer understanding of hand-altered attention as a product of both the static and dynamic features of the hands working in conjunction. The question of coordination in visual attention also applies to social contexts, as when two people work together on a jigsaw puzzle. What role do *other people's* hands play in shaping how people allocate their own attentional resources? Thanks to Sun and Thomas (2013), we now understand that people can and do prioritize the space near a friend's hand following a collaborative joint-action task.

## Visual perception near the hands: biases and theories

As a field, we have made significant progress into understanding what HAV is and how it works by documenting the variety of mental processes that are affected by hand-proximity. Quite often, the reported effects have taken the form of biases or tradeoffs in visual processing that correspond with relative hand placement. The utility of identifying these biases lies in what they can tell us about the neural mechanisms that give rise to HAV.

Two studies in this Topic report processing biases that imply a strong right-hemisphere involvement in near-hand effects. First, Langerak et al. (2013) show that people preferentially process global vs. local information near their left hand but not their right. Second, in a study that considers near-hand effects on auditory processing, Tseng et al. (2014) find that hand-proximity elicits faster tone localization to the left, with no such advantage to the right. In both cases, evidence of right-hemisphere involvement supports the *parietal lobe account* of HAV, which attributes near-hand effects to (right) parietal mechanisms involved in multisensory integration and body-space coding. Such evidence is also consistent with the newly emerging *magnocellular account* of HAV. According to this account, hand-proximity biases visual processing along the action-oriented magnocellular dorsal pathway, which incidentally also includes the parietal lobe and favors motion, location, and low spatial frequency (LSF) information over color, detail, and high SFs. It follows, then, that people ought to be better at LSF tasks near their hands, and Chan et al. (2013) show evidence of this through enhanced gist processing. It also follows that magnocellular biases ought to be reflected in how people remember information near their hands. In support of this, Kelly and Brockmole (2014) report a dissociation in people's working memory capacity for orientation (+) vs. color (−) information that corresponds to hand-proximity.

Theories of HAV generally agree that the purpose of HAV is to facilitate interaction with the environment. This is consistent with other research literatures that have also found evidence of specialized mechanisms for supporting interaction. As one example, perceiving visual content that contains action-relevant information primes the motor system for action. Here, Wilf et al. (2013) show that this effect is not purely cognitive nor driven by top-down biases, but rather can be detected in the muscles via electromyography at early stages of movement execution. As a second example, tools can become incorporated into the body schema at the cognitive and neural level. Furthermore, the space near the functional end of a tool is awarded many of the same processing advantages as near-hand space. Here, Brown and Goodale (2013) review the literature on near-tool effects, and they conclude that motor knowledge is critical for these effects to emerge.

In considering HAV in the context of affordances, tool-use, and the like, we notice something akin to a cycle. HAV helps us perceive action-relevant information in the environment; perceiving action-relevant information primes us to take action; if taking action results in taking possession of and wielding a tool, the body schema adapts accordingly, and visual processing of near-tool space is altered to facilitate interaction; and so on.

### Conflict of interest statement

The authors declare that the research was conducted in the absence of any commercial or financial relationships that could be construed as a potential conflict of interest.
